# *mTOR* mutation disrupts larval zebrafish tail fin regeneration via regulating proliferation of blastema cells and mitochondrial functions

**DOI:** 10.1186/s13018-024-04802-z

**Published:** 2024-05-29

**Authors:** Gongyi Xiao, Xiangwei Li, Huiping Yang, Ruobin Zhang, Junlan Huang, Yu Tian, Mao Nie, Xianding Sun

**Affiliations:** 1 Center for Joint Surgery, Department of Orthopedic Surgery, The Second Affiliated Hospital of Chongqing Medical University, 74 Linjiang Road, Chongqing, 400010 China; 2 Department of Cardiology, The Second Affiliated Hospital of Chongqing Medical University, 74 Linjiang Road, Chongqing, 400010 China; 3 Department of Wound Repair and Rehabilitation Medicine, State Key Laboratory of Trauma, Burns and Combined Injury, Trauma Center, Research Institute of Surgery, Daping Hospital, Army Medical University, Chongqing, 400042 China; 4 Ministry of Education College of Bioengineering, Chongqing University, Chongqing, 400044 P. R. China

**Keywords:** *mTOR*, Zebrafish, Larvae fin, Regeneration, Mitochondrial fission, Ca^2+^

## Abstract

**Background:**

The larval zebrafish tail fin can completely regenerate in 3 days post amputation. *mTOR*, the main regulator of cell growth and metabolism, plays an essential role in regeneration. Lots of studies have documented the role of *mTOR* in regeneration. However, the mechanisms involved are still not fully elucidated.

**Materials and results:**

This study aimed to explore the role and mechanism of *mTOR* in the regeneration of larval zebrafish tail fins. Initially, the spatial and temporal expression of *mTOR* signaling in the larval fin was examined, revealing its activation following tail fin amputation. Subsequently, a *mTOR* knockout (*mTOR*-KO) zebrafish line was created using CRISPR/Cas9 gene editing technology. The investigation demonstrated that *mTOR* depletion diminished the proliferative capacity of epithelial and mesenchymal cells during fin regeneration, with no discernible impact on cell apoptosis. Insight from SMART-seq analysis uncovered alterations in the cell cycle, mitochondrial functions and metabolic pathways when *mTOR* signaling was suppressed during fin regeneration. Furthermore, *mTOR* was confirmed to enhance mitochondrial functions and Ca^2 +^ activation following fin amputation. These findings suggest a potential role for *mTOR* in promoting mitochondrial fission to facilitate tail fin regeneration.

**Conclusion:**

In summary, our results demonstrated that *mTOR* played a key role in larval zebrafish tail fin regeneration, via promoting mitochondrial fission and proliferation of blastema cells.

**Supplementary Information:**

The online version contains supplementary material available at 10.1186/s13018-024-04802-z.

## Introduction

Regenerative medicine, especially epimorphic regeneration, aims to explore the repair of organ and tissue defects in the body, as well as the regeneration and functional reconstruction of defective organ tissues. The regenerative capacity of many vertebrates, including humans, is much weaker compared with that of invertebrates, caudate amphibians, and Osteichthyes [1]. Therefore, a comprehensive understanding of the molecular mechanisms for regeneration is essential to improve the fight against tissue defects, aging and various diseases.

Zebrafish, an animal model extensively used in various scientific researches, exhibits remarkable regenerative capabilities [2]. Notably, the zebrafish fin serves as an exceptional model for regenerative medicine, due to its rapid and complete regeneration, and the convenience of establishing a fin regeneration model. Zebrafish larvae and adults have conserved fin regeneration mechanisms [3]. Besides, larval fin cells are at an early stage of undifferentiated state. In addition, fin of zebrafish larvae has a simpler structure compared with that of adults, consisting mainly of epithelial cells, mesenchymal cells and hematopoietic cells [4]. Since the establishment of the larval fin regeneration model [3], it has gained increasing attraction among researchers. Previous studies have identified numerous key genes involved in regeneration, along with associated signaling pathways and other influencing factors such as inflammation and macrophages [5, 6]. However, there is still no a definitive conclusion. With the advancement of sequencing and genealogical tracing technologies, the role of mitochondria in regeneration has garnered increasing attention [7]. The regeneration process demands higher energy levels than the normal metabolic state, making mitochondria an apparent cornerstone of regeneration.

Mechanistic target of rapamycin (*mTOR*), a serine / threonine kinase, was first identified in 1964 [8]. It has been identified as a target of rapamycin in yeast and is widely expressed in most eukaryotes [9–11]. As a key regulator of cellular metabolism, *mTOR* participates in the regeneration of a wide range of tissues and organs, such as the heart, liver, retina, nerves, muscles and fins [12]. Hirose et al. [13] demonstrated that *mTOR* regulated the proliferation, survival, and differentiation of blastema cells, wound epidermal cells and osteoblasts downstream of IGF and Wnt signaling pathways during different stages of fin regeneration. A recent study identified the *mTORC1* pathway as a crucial upstream signal mediating tissue regeneration in the axolotl [14]. However, the underlying regulatory mechanisms remain to be elucidated.

To address this issue, we constructed *mTOR* knockout (*mTOR*-KO) fish line using CRISPR/Cas9 gene editing technology and established a larval zebrafish tail fin regeneration model. Our findings indicate that following fin amputation in zebrafish larvae, *mTOR* is activated and involved in fin regeneration. Genetic knockout of *mTOR* reduces mitochondrial fission, blastema cells proliferation and Ca^2+^ signaling activation post-amputation, leading to impaired fin regeneration.

## Methods

### Zebrafish strains and generation of transgenic lines

The AB and transgenic zebrafish lines employed in this study were obtained from the Zebrafish Resource Center of China. All zebrafish were housed in semi-closed recirculation housing systems (ESEN, China) and were kept at a constant temperature (28.5 °C) on a 14 h:10 h light: dark photoperiod. All zebrafish lines were raised and maintained in line with standard protocols [15]. All in vivo experiments and protocols were approved by Institutional Animal Care and Use Committee of the Research Institute of Surgery, Daping Hospital IACUC protocol SYXK- (Army) 2022-0003.

### Zebrafish *mTOR* knockout, gene extraction and identification

The *mTOR*-KO zebrafish line was established using CRISPR/Cas9 gene editing technology. We designed the knockout target at 4th exon of *mTOR*. The process was performed as previous described [16]. The *mTOR* target sequence was 5’-GGAGATGGCCTCTAAAGCCA-3’, and the target region was amplified by PCR using the following primers: forward primer: 5’- GTGAGCCTGATTGGAGTGGAG-3’; reverse primer: 5’-GATCTACGCACCGCTGCATG-3’.

Gene extraction and identification: tail fin tissues were cut into a 200 µl PCR tube, 10–50 µl 50 mM NaOH was added into the tube and then subjected to PCR system at 95°C for 20–30 min, 4°C hold heating lysis of embryos, followed by addition of 1/10 volume of 1 M Tris-HCl (pH 8.0). The contents were vortexed and shaken. 2 µl of the sample was subjected to PCR amplification (25 µl system: ddH2O 19.25 µl, 10Xbuff 2.5 µl, dNTP 1µl; 5’ primer 0.5 µl, 3’ primer 0.5 µl, rTaq enzyme 0.125 µl). 20 µl PCR product was collected and mixed with 5 µl of the loading buffer (6×) and then transferred to a 2% agarose gel containing GoldenView at 160 V for 20 min and imaged using a gel imager (#GelDoc2000, Bio-Rad, USA).

### Whole-mount in situ hybridization (ISH) analysis

Sequences of respective genes that cover 0.4-1.0 kb of coding sequences were PCR-cloned. Later, PCR products were cloned into the pGEMT-easy vector (Promega). Digoxigenin-labeled probes were generated by in vitro transcription (DIG RNA Labeling Kit, Roche, America) and ISH was carried out according to the zebrafish book. Briefly, embryos were fixed overnight at 4 °C in 4% paraformaldehyde (PFA), dehydrated through a methanol series and stored overnight at − 20 °C. Subsequently, the embryos were rehydrated in PBST (PBS + 0.1% Tween 20) and pre-hybridized for 2–5 h at 68.5 °C in hybridization solution containing 50% formamide. Hybridization was performed with 0.5-1 ng/µl antisense probes diluted in hybridization solution overnight at 68.5 °C. This was followed by washing with 2× SSCT buffer and blocking in 1% blocking reagent (#11,096,176,001, Roche, America) at room temperature for 1 h. The embryos were then incubated with anti-Dig-AP antibody (dilution 1: 5000, #11,093,274,910, Roche, America) at 4 °C overnight. Embryos were rinsed extensively for 3 h, and then stained with NBT/BCIP solution (dilution 1: 50, #11,681,451,001, Roche, America). The WISH images were captured using a SteREO Discovery 20 microscope (Carl Zeiss, Germany). The primers used for ISH were designed using SnapGene and checked by Oligo 7. Relevant primer sequences are listed in the Supplementary Table 1.

### Antibody staining

Whole mount antibody staining was performed following the zebrafish book with minor modifications. Briefly, embryos were fixed in 4% formaldehyde, washed with 0.3% Triton X-100 in PBS, and blocked in the blocking solution (4% BSA combined with 0.3% Triton X-100 in PBS) at 4 °C for 2 h. The primary antibodies of rabbit anti-pS6 (S235/236) (dilution 1:300, #4858, Cell Signaling Technology, America), rabbit anti-S6 (dilution 1:300, #2217, Cell Signaling Technology, America) were diluted in the blocking solution and incubated at 4 °C overnight. The embryos were then washed with 0.3% Triton X-100 in PBS and incubated with secondary antibodies donkey-anti-Rabbit Alexa Fluor 568 (dilution 1:500, #A10042, Invitrogen, America) at 37 °C for 30 min. They were washed 4–8 times, and then imaged using LSM880NLO confocal microscope (Carl Zeiss, Germany).

### Edu cell proliferation assay and TUNEL staining

S-phase labeling was performed using the Edu cell proliferation assay according to manufacturer’s instructions. At 24 h and 48 h post amputation (hpa), embryos were injected with 0.2 mM Edu (#E131265, Aladdin, Shanghai, China) and incubated at 28.5 °C for 30 min. After overnight fixation in 4% PFA at 4 °C, the embryos underwent the Edu assay. Apoptosis in the regenerating fin at 24 hpa was labeled using in situ Cell Death Detection kit TMR Red (#12,156,792,910, Roche, America), following the manufacturer’s protocol.

### Drug treatment

Torin1 (#1222998-36-8, MCE, Shanghai, China) and Rapamycin (#HY-10,219/CS-0063, Shanghai, China) were dissolved in DMSO. A reserve solution of 1 mM for Torin1 and 2 mM for Rapamycin was prepared and stored at -20 °C. After fertilization, Torin1 was diluted to 500 nM and Rapamycin to 1 μm, and the developing embryos were treated from 2 days post fertilization (dpf). Those in the control group were treated with the same dose of DMSO, and then reformulated and replaced every day until the end of the experiment to ensure sustained treatment effects.

### Mito-Tracker and JC-1 staining

Mito-Tracker (#C1049B, Beyotime, Shanghai, China) staining: 3 dpf larvae were cultured in E3 medium (5 mM NaCl, 0.17 mM KCl, 0.33 mM CaCl_2_, 0.33 mM MgSO4) containing 5 µM Mito-Tracker Red (Molecular Probes) for 2 h. They were subsequently washed three times before imaging. To examine the mitochondrial morphology, the larvae were stained with the Mito-Tracker Red and then subjected to tail fin amputation and then fixed with low melting point agarose for confocal imaging.

For the JC-1 (#C2006, Beyotime, Shanghai, China) staining: 3 dpf larvae were cultured in JC-1 working solution (followed by the instructions) for 2 h. They were then washed three times and imaged. The subsequent steps were similar to those used for Mito-Tracker.

### SMART-seq

Sequencing samples were obtained from fin tissues at 24 hpa. Fifty regenerated fins were collected from the control and experimental groups that treated with 1 µM Rapamycin. Three biological replicates were prepared for each group and the samples were lysed using Trizol. Total RNA was extracted and used to synthesize cDNA with SMARTScribe™ Reverse Transcriptase. Next, cDNA was fragmented by dsDNA fragmentase by incubating at 37 °C for 30 min. The ligated products were amplified using PCR to construct libraries, and paired-end sequencing was performed on the illumina Novaseq™ 6000 platform by LC Bio Technology CO.Ltd (Hangzhou, China). The differentially expressed genes (DEGs) between control group and experimental group were determined using the threshold of |log_2_FC|>=1 and *P* < 0.05. To explore the potential mechanism by which *mTOR* regulates fin regeneration, GO, KEGG and GSEA enrichment analysis were performed.

### RNA isolation and quantitative PCR (qPCR) analysis

100 regenerated fin tissues from *mTOR*-WT and *mTOR*-KO zebrafish larvae at 48 hpa were collected, and crushed using an ultra-low-temperature grinder (#KZ-5 F-3D, Servicebio, Wuhan, China), and RNA was extracted using the Trizol method to obtain approximately 5 µg of RNA. Total RNA was reverse transcribed into cDNA and then subjected to qPCR following the instructions on the kit (#RR820A, Takara, Japan). Relevant primer sequences used for qPCR are listed in the Supplementary Table 2.

### GCamp6s lives imaging

Zebrafish larvae at 3 dpf underwent fin amputation and were subsequently immobilized in a confocal dish using low-melting-point agarose. The live stained larvae were then imaged every second using an a LSM880NLO confocal microscope (Carl Zeiss, Germany).

### ROS production assay

Zebrafish larvae at 3 dpf were incubated in egg water containing 20 µM Dichloro-dihydro-fluorescein diacetate (DCFH-DA, #S0033S, Beyotime, Shanghai, China) for 30 min at 28.5 °C in the dark. Then, they were washed in egg water, later anesthetized using 0.2% Tricaine (#A5040, Sigma-Aldrich, America) and immediately examined under a fluorescence microscope (Carl Zeiss, Germany). The fluorescence intensity of the fin area was analyzed using Image J.

### Transmission electron microscope

The morphology and size of mitochondria were examined using a electron microscope (TEM). Larval zebrafish tail fin at 6 hpa were collected and then were immediately fixed in 2.5% glutaraldehyde (#G1102, Servicebio, Wuhan, China) at 4 °C for 24 h. Then they were dehydrated, embedded, sectioned, stained and visualized using a Hitachi HT7800 transmission electron microscope (Hitachi, Tokyo, Japan).

### Statistical analysis

All numeric data were presented as the mean ± SD. Error bars indicate SD. Differences between two groups were compared using Unpaired Student’s t test, and ANOVA was employed in the comparison of multiple groups. When significant levels (*P* < 0.05) were achieved, Tukey’s Post Hoc test was performed. All statistical analyses were performed using GraphPad PRISM 9.0 software. *P* < 0.05 was considered statistically significant.

## Results

### *mTOR* signaling was activated in zebrafish larvae after tail fin amputation

**Fig. 1:**
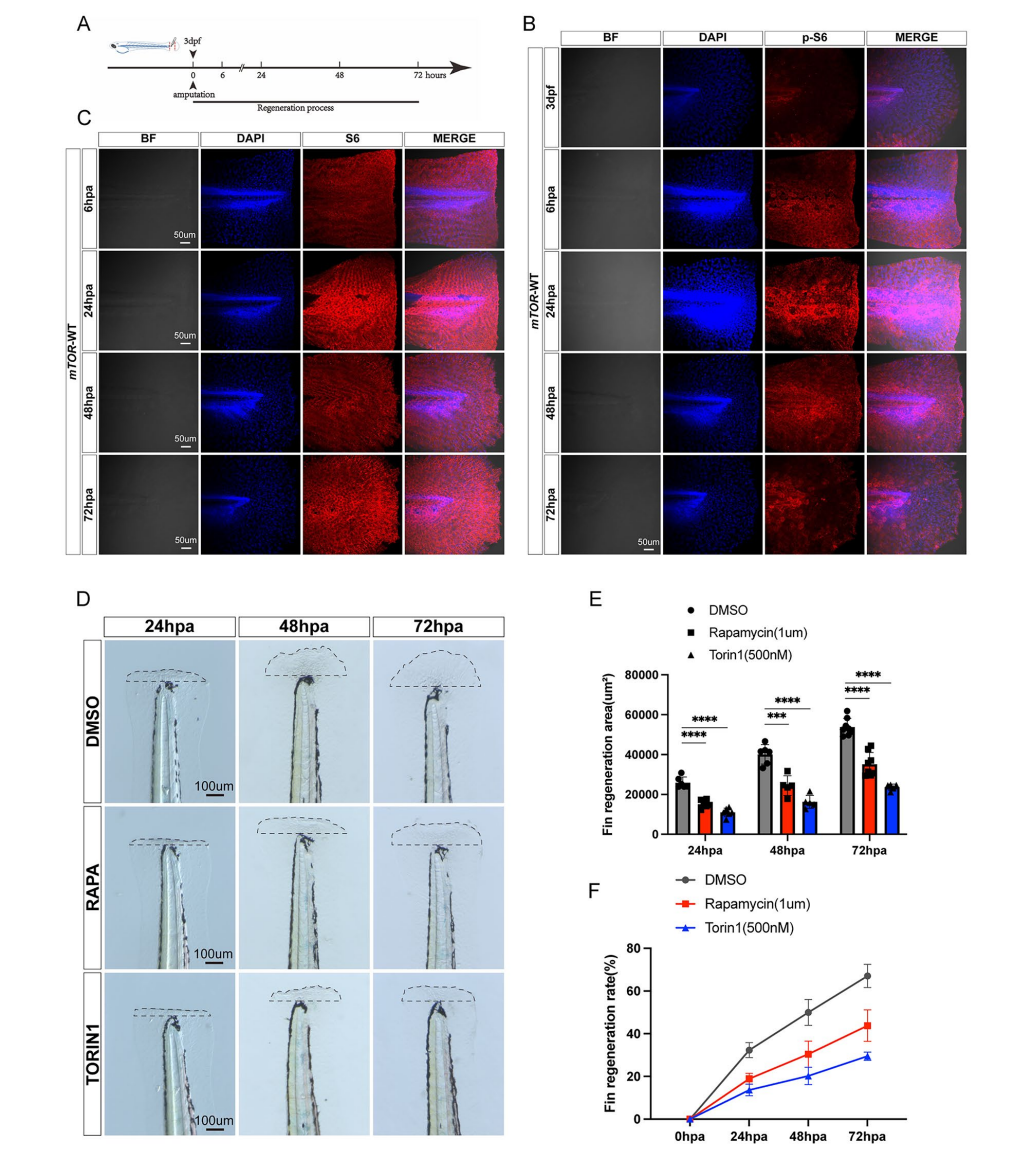
mTOR signaling was activated in larval zebrafish tail fin after amputation. (**A**) Fin amputation model: the tail fin was amputated at 3 dpf. (**B**) The expression level of p-S6 in *mTOR*-WT larval zebrafish fin at different stages after amputation. (**C**) The expression level of S6 in *mTOR*-WT larval zebrafish fin at different stages after amputation. (**D**) Effect of *mTOR* inhibitors (Rapamycin and Torin1) on larval zebrafish fin regeneration after amputation. (**E-F**) Statistical analysis of fin regeneration area and rate in *mTOR*-WT zebrafish larvae after administration of Rapamycin and Torin 1. ***P < 0.001, ****P < 0.0001

The investigation into the role of *mTOR* in larval fin regeneration involved the amputation of the tail fin at 3 dpf to establish a fin regeneration model (Fig. 1A). Antibody staining was employed to detect *mTOR* signaling before and after amputation. The expression level of p-S6 exhibited a gradual increase from 0 hpa to 48 hpa, followed by a decrease from 48 hpa to 72 hpa (Fig. 1B**).** Conversely, the expression of S4 remained stable in the fins (Fig. 1C). The experiments conducted in our study demonstrated the activation of *mTOR* signaling following fin amputation, aligning with previous research findings on adult zebrafish fin regeneration [13]. Next, to investigate whether *mTOR* was required for larval fin regeneration, we employed different *mTOR* inhibitors, rapamycin and Torin1, to analyze the regeneration process of larval zebrafish fin. We recorded histomorphological photographs of the regenerated fin at 24 h, 48 h and 72 h respectively after amputation, and found that the tail fin in the control group could reach nearly complete regeneration at 72 hpa, whereas the fin in the group treated with *mTOR* inhibitors could not reach a fully regeneration (Fig. 1D). Further tests showed that the area of the regenerated fin in the inhibitor treated group was significantly smaller relative to that in the control group (Fig. 1E). In addition, the fin regeneration rate of the inhibitor group was significantly lower compared with that of the control group (Fig. 1F). In conclusion, these experiments suggested that *mTOR* signaling was activated after fin amputation, while inhibition of *mTOR* suppressed the fin regeneration area and rate.

### *mTOR* knock out attenuated zebrafish larval fin regeneration

**Fig. 2:**
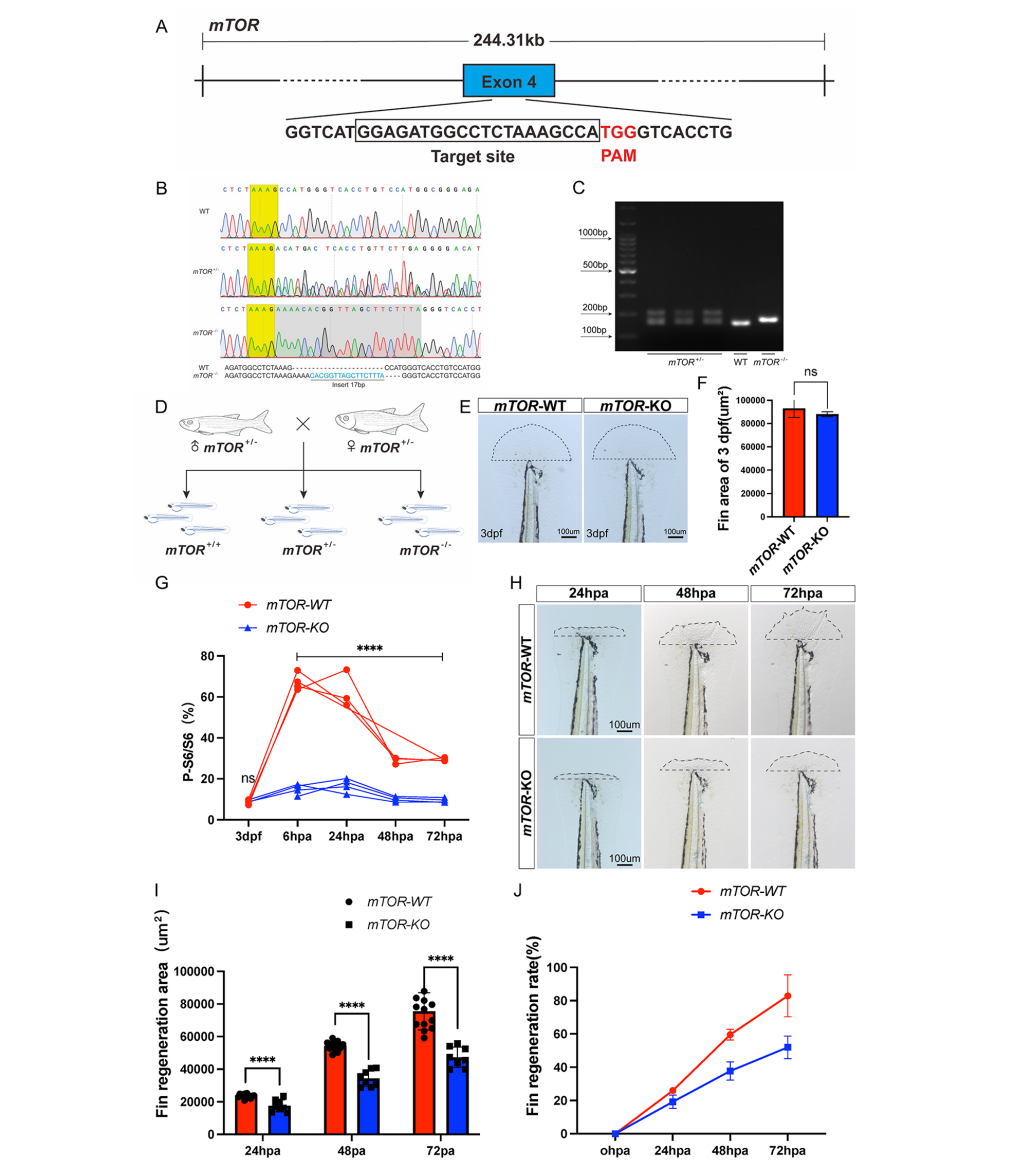
Construction of *mTOR* knock out zebrafish using CRISPR/Cas9 technology. (**A**) The gRNA targeted sequences in the exon 4 of *mTOR* gene. (**B**) Sanger sequencing results after injection of Cas9 mRNA. (**C**) DNA gel electrophoresis was used to detect PCR products. (**D**) Schematic diagram showing the screening of *mTOR*^−/−^ fish line through Self-fertilization. (**E-F**) The fin area of un-amputated *mTOR*-WT and *mTOR*-KO zebrafish at 3 dpf. (**G**) Statistical analysis of p-S6/S6 between *mTOR*-WT and *mTOR*-KO larval zebrafish fin after amputation. (**H**) Effect of *mTOR* knock out on larval zebrafish fin regeneration after amputation. (**I-J**) Statistical analysis of fin regeneration area and rate after fin amputation between *mTOR*-WT and *mTOR*-KO zebrafish larvae. ^ns^*P* > 0.05, ****P < 0.0001

To further clarify the impact of *mTOR* in larval zebrafish fin regeneration, we constructed *mTOR*-KO fish line using CRISPR/Cas9 gene edit technology. Exon 4 of the *mTOR* gene was chosen to design gRNA site, and the PAM region was TGG. The target site was GGAGATGGCCTCTAAAGCCA (Fig. 2A). Sequencing analysis uncovered distinct patterns for the *mTOR*^+/−^ and mTOR-/- genotypes. The *mTOR*^+/−^ genotype exhibited evident overlapping peaks, while the *mTOR*^−/−^ genotype displayed single peaks with varied sequences compared to *mTOR*-WT. Comparison results revealed the insertion of 17 bases into the *mTOR*^−/−^ genotype, as indicated by the blue-labeled sequence (Fig. 2B). Agarose gel electrophoresis of PCR products showed that *mTOR*^+/−^ genotype was two bands, *mTOR*-WT was a single band with smaller molecular weight, and *mTOR*^−/−^ genotype was a single band with larger molecular weight (Fig. 2C). *mTOR*^+/−^ self-fertilization was performed to establish the *mTOR*^−/−^ fish line (Fig. 2D).

*mTOR* is a key regulator in protein synthesis, cell growth and proliferation. In the present investigation, it was noted that *mTOR* knock out resulted in the abnormal formation of the swimming bladder in zebrafish, while having no discernible impact on the development of the larval fin (Supplementary Fig. 1A). Results showed that there was no significant difference in fin area between *mTOR*-WT and *mTOR-KO* zebrafish larvae at 3 dpf and 6 dpf (Fig. 2E-F, Supplementary Fig. 1B-C). Next, the larvae fin was amputated, and the perpendicular line to the end of the notochord was selected as the amputation site for modeling (Supplementary Fig. 1D). The results showed no difference in the expression of S6 and p-S6 between *mTOR*-WT and *mTOR*-KO zebrafish at 3 dpf (Supplementary Fig. 1E-F). Following this, we conducted quantitative analysis of S6 and p-S6 expression at various time intervals post tail fin amputation (Supplementary Fig. 1G-H). Our findings revealed that the *mTOR* signaling pathway did not exhibit activation following amputation in *mTOR*-KO zebrafish larval fin. Quantitative analysis indicated that p-S6/S6 expression level in the *mTOR* knock out zebrafish was nearly at the baseline level (Fig. 2G). These results indicated that the *mTOR*-KO fish line was successfully constructed.

Similar to the results obtained following administration of *mTOR* inhibitors, *mTOR* knock out suppressed the larval zebrafish fin regeneration (Fig. 2H). The fin regeneration area and rate in *mTOR*-KO zebrafish were lower compared with those in the *mTOR*-WT group (Fig. 2I-J**).**

### *mTOR* promoted epithelial and mesenchymal cells proliferation during larval zebrafish fin regeneration

Following fin amputation, the stump promptly seals and initiates the formation of wound epithelium and blastema tissues within a span of 12 h. Subsequently, the larval fin undergoes regeneration through the proliferation of blastema-like cells [3]. In our experiments, inhibiting *mTOR* attenuated regeneration, but blastema tissues were still formed. We speculated that *mTOR* could still regulate blastema cell cycle during the regenerative growth phase after blastema formation.

**Fig. 3:**
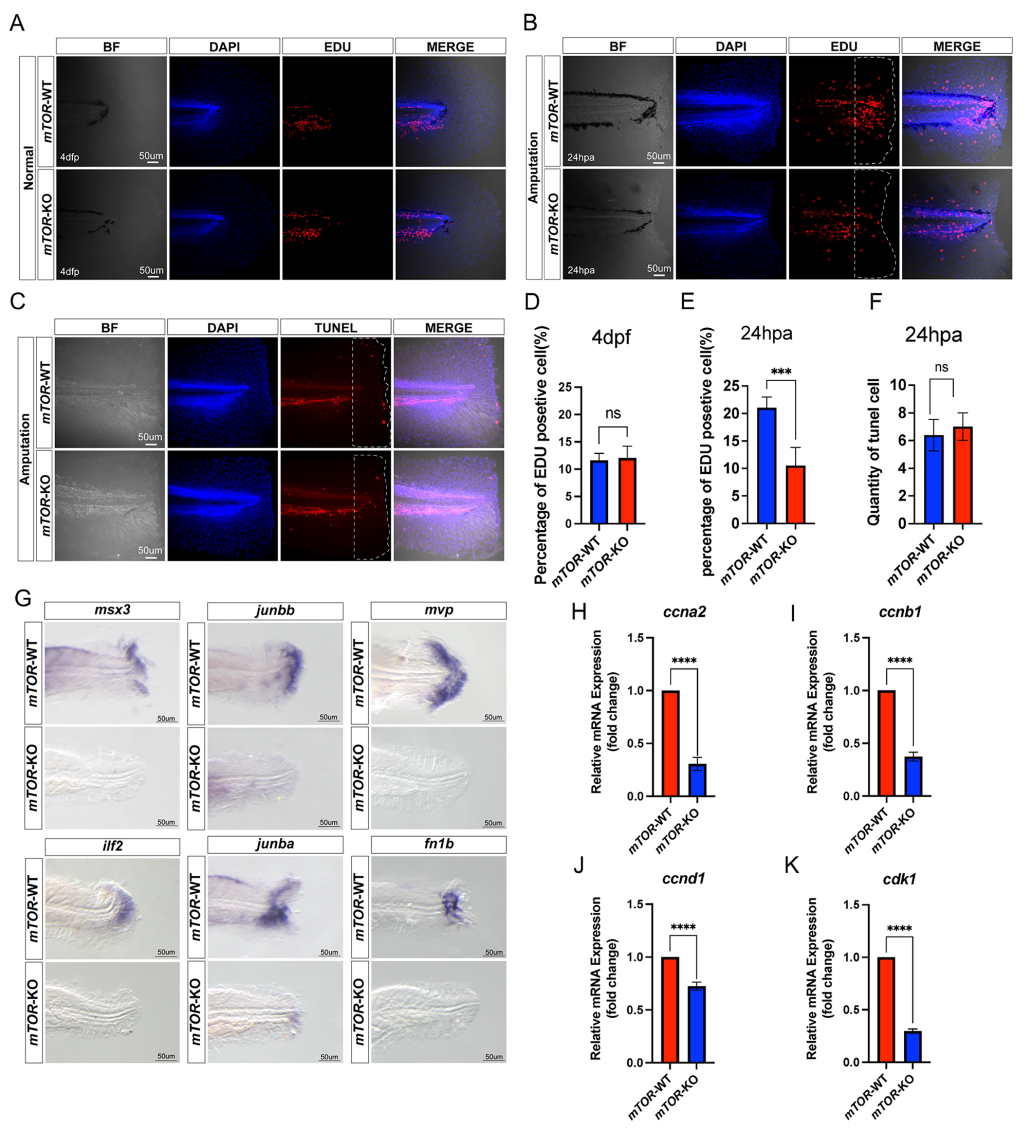
mTOR promoted epithelial and mesenchymal cells proliferation during larval zebrafish fin regeneration. (**A, D**) EDU staining of *mTOR*-WT and *mTOR*-KO larval zebrafish fin at 4 dpf. (**B, E**) EDU staining of *mTOR*-WT and *mTOR*-KO larval zebrafish fin at 24 hpa. (**C, F**) TUNEL staining of *mTOR*-WT and *mTOR*-KO larval zebrafish fin at 24hpa. (**G**) Location of *msx3*, *junbb*, *mvp*, *ilf2*, *junba*, *fn1b* in *mTOR*-WT and *mTOR*-KO larval zebrafish fin by in situ hybridization at 48 hpa. (**H-K**) The mRNA expression levels of cell cycle-related molecules (*ccna2*, *ccnb1*, *ccnd1* and *cdk1*) between *mTOR*-WT and *mTOR*-KO larval zebrafish fin at 48 hpa. ^ns^*P* > 0.05, ***P < 0.001, ****P < 0.0001

Initially, we measured the proliferation and apoptosis of blastema cells using EDU and TUNEL staining assays, respectively (Fig. 3A-C; Supplementary Fig. 2A, C). We found that there was no significant difference in the proliferation of blastema cells between the *mTOR*-WT and *mTOR*-KO zebrafish fin in the unamputated state (Fig. 3D, Supplementary Fig. 2B). After fin amputation, the proliferation of blastema cells in the *mTOR*-KO group was significantly lower relative to that of the *mTOR*-WT group (Fig. 3E, Supplementary Fig. 2D), whereas apoptosis was not significantly different between the two groups (Fig. 3F).

In order to discern the particular cell type within the blastema whose proliferation was influenced, we developed specific labeling probes targeting distinct cell populations. Subsequently, we utilized zebrafish whole-mount in situ hybridization to assess the expression levels of various cell types throughout the blastema formation phase. (Fig. 3G). The results showed that the expression levels of *msx3*, *junbb*, *mvp*, and *ilf2* which labeled mesenchymal cells as well as *junba* and *fn1b* which labeled epithelial cells was significantly downregulated in the *mTOR*-KO group. In addition, the mRNA expression of cell cycle- related genes (*ccna2*, *ccnb1*, *ccnd1* and *cdk1*) was significantly lower in the *mTOR*-KO group relative to that in the *mTOR*-WT group (Fig. 3H-K). Altogether, these findings demonstrated that *mTOR* promoted the proliferation of epithelial and mesenchymal cells in the blastema tissues during fin regeneration without affecting the cell apoptosis.

### SMART-seq analysis revealed changes in immune response, cell cycle and mitochondrial functions

**Fig. 4:**
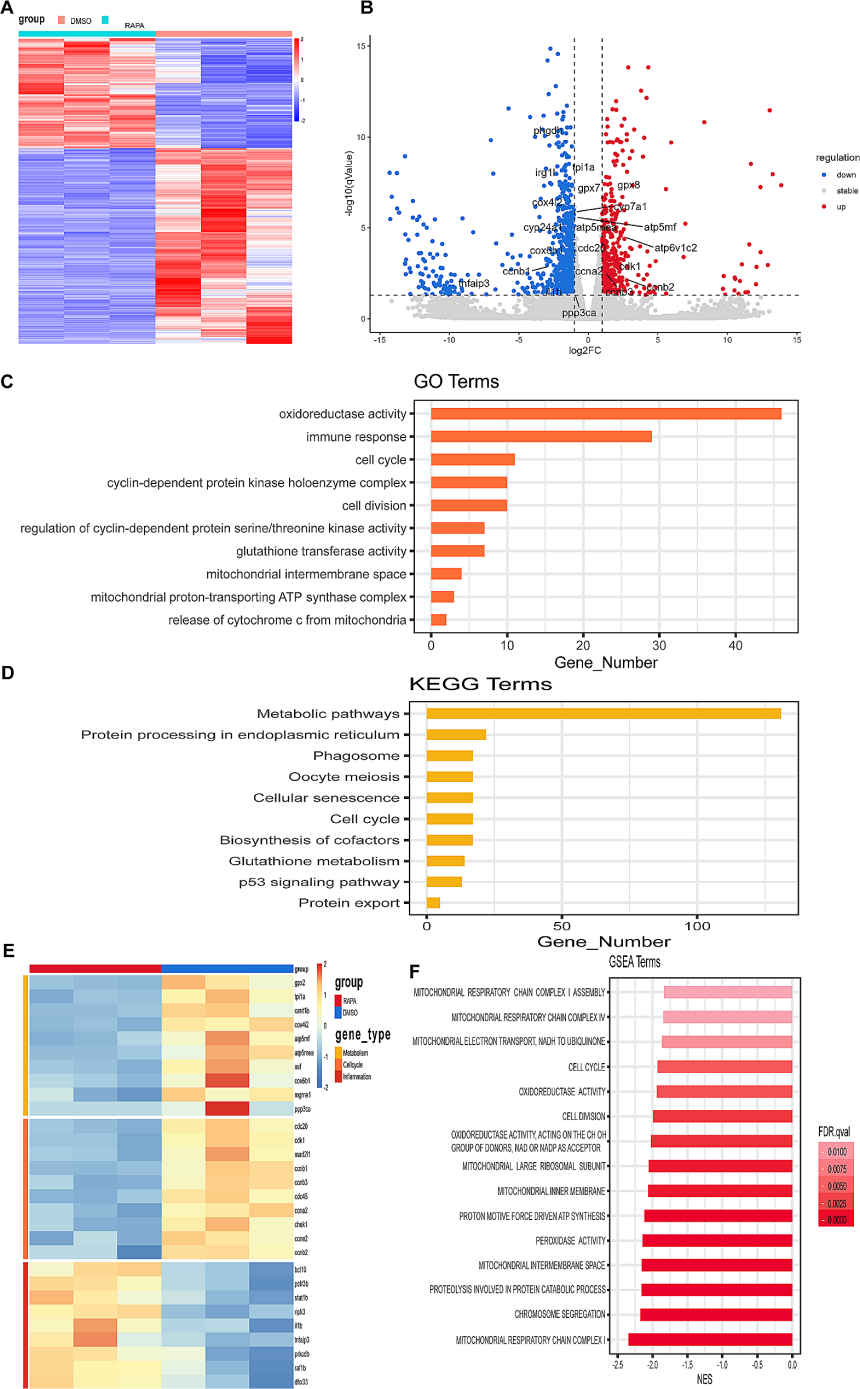
SMART-seq results of regenerated fins between *mTOR*-WT and rapamycin treated zebrafish larvae. (**A**) Heatmap of DEGs between *mTOR*-WT and rapamycin treated larval zebrafish tail fin. (**B**) Volcano map of DEGs between *mTOR*-WT and rapamycin treated larval zebrafish fin. (**C**) GO analysis of DEGs between the two groups. (**D**) KEGG pathway analysis of DEGs between the two groups. (**E**) Heatmap of metabolism, cell cycle and inflammation related genes between the two groups. (**F**) GSEA enrichment analysis of DEGs

In order to investigate the potential mechanisms through which *mTOR* regulates the regeneration of larval zebrafish tail fins, we obtained regenerated fins from larval zebrafish at 24 hpa that were either treated with or without rapamycin. These were then subjected to SMART-seq analysis, with each group consisting of 3 replicates. The results showed that there were totally 1352 differentially expressed genes (DEGs) between *mTOR*-WT and rapamycin treated zebrafish (Fig. 4A), among which 487 genes were upregulated and 865 genes were downregulated (Fig. 4B). The gene ontology (GO) analysis revealed a notable enrichment of DEGs in several key functional categories, including immune response, cell cycle regulation, as well as mitochondrial intermembrane space and mitochondrial proton-transporting ATP synthase complex(Fig. 4C). KEGG pathways analysis showed that metabolic pathways, cell cycle were altered in DEGs (Fig. 4D). Heatmap of DEGs showed that metabolism, cell cycle and inflammation associated genes were different between the two groups (Fig. 4E). GSEA enrichment analysis indicated that mitochondrial respiratory chain complex, mitochondrial large ribosomal subunit, mitochondrial intermembrane space and mitochondrial inner membrane were highly enriched in DEGs (Fig. 4F). Therefore, we speculated that *mTOR* regulated larval zebrafish fin regeneration via regulating mitochondrial functions.

### *mTOR* knock out inhibited mitochondrial fission during fin regeneration

**Fig. 5:**
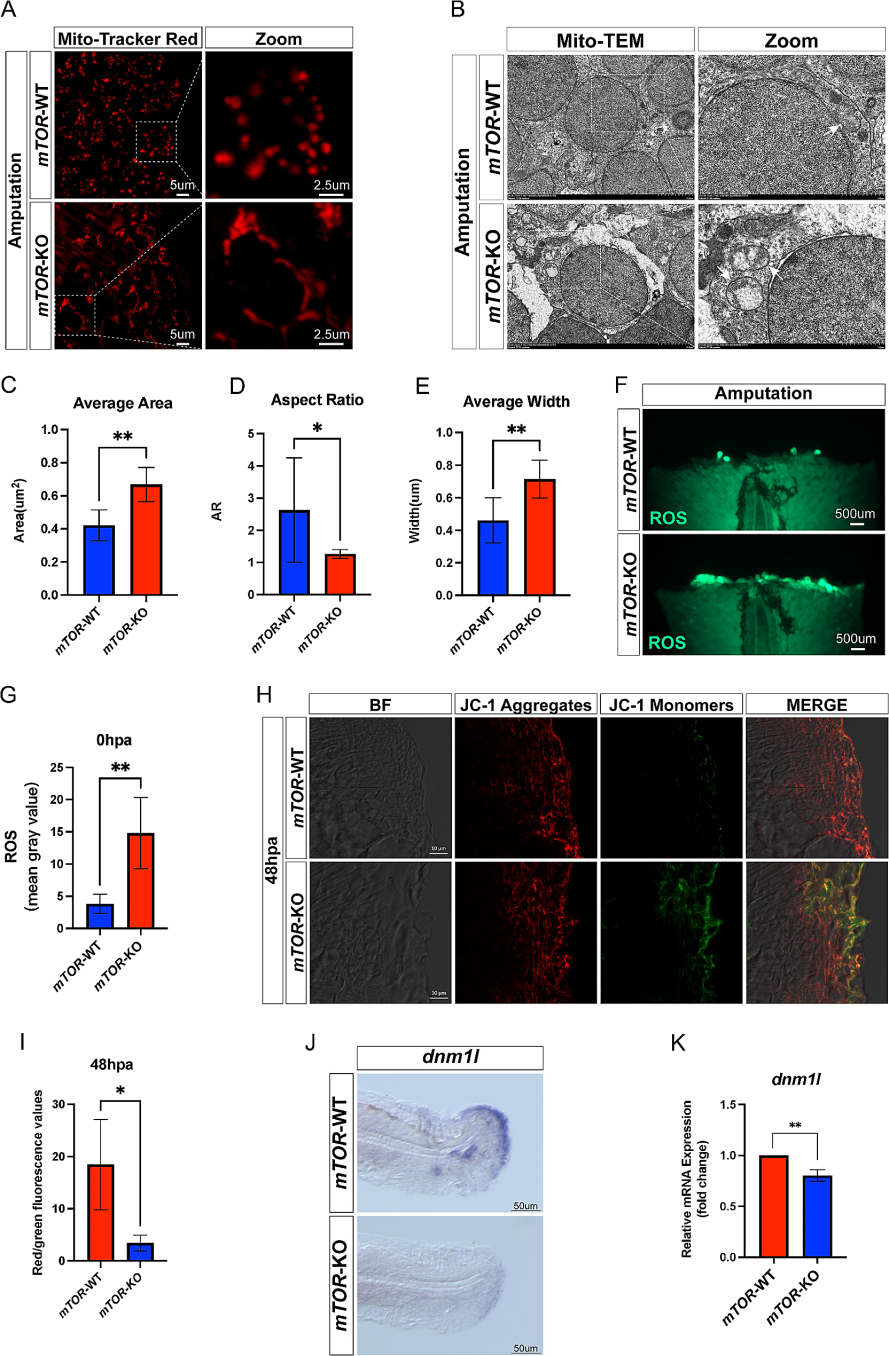
mTOR knock out affected mitochondrial morphology and membrane potential. (**A**) Mito-Tracker red staining of *mTOR*-WT and *mTOR*-KO larval zebrafish fin following amputation. (**B**) Representative transmission electron microscope images of *mTOR*-WT and *mTOR*-KO larval zebrafish fin after amputation. (**C-E**) Statistical analysis of the average area, aspect ratio and average width of mitochondria between *mTOR*-WT and *mTOR*-KO larval zebrafish fin after amputation. (**F-G**) The ROS levels between *mTOR*-WT and *mTOR*-KO larval zebrafish fin after amputation. (**H-I**) Measurement of the mitochondrial membrane potential by JC-1 staining between *mTOR*-WT and *mTOR*-KO zebrafish at 48 hpa. (**J**) Location of mitochondrial fission related gene (*dnm1l*) in *mTOR*-WT and *mTOR*-KO larval zebrafish fin by in situ hybridization. (**K**) mRNA expression level of *dnm1l* in *mTOR*-WT and *mTOR*-KO larval zebrafish fin. *P < 0.05, **P < 0.01

To confirm the role of *mTOR* in mitochondrial functions, we measured the expression of *cox6b1*, which is involved in electron transport in the mitochondrial respiratory chain. RT-qPCR results showed that *cox6b1* was down-regulated in the *mTOR*-KO zebrafish compared with that in the *mTOR*-WT group after amputation (Supplementary Fig. 3A). Subsequently, we investigated the mitochondrial morphology in zebrafish larvae before and after fin amputation using Mito-Tracker red staining. The results illustrated that both the *mTOR*-WT and *mTOR*-KO groups displayed elongated signals within the mitochondria when unamputated (Supplementary Fig. 3B). Post-amputation, the *mTOR*-WT group exhibited patchy split signals, while the *mTOR*-KO group displayed partial punctate split signals and elongated stripes. (Fig. 5A). During structural regeneration, mitochondrial fragmentation was increased in the amputated fins [17]. TEM results observed the mitochondrial fission in *mTOR*-WT group, while the mitochondria were swollen and degenerated, with whitish areas inside in the *mTOR*-KO group (Fig. 5B). Quantitative analysis indicated that the average area and width in the *mTOR-KO* group were significantly higher than those of the *mTOR*-WT group (Fig. 5C, E), while the aspect ratio was decreased (Fig. 5D). Mitochondrial dysfunctions usually accompanied by ROS accumulation. In our study, the ROS levels were elevated in the *mTOR*-KO zebrafish compared with that in the *mTOR*-WT zebrafish after amputation (Fig. 5F-G). Moreover, the mitochondria membrane potential in the *mTOR*-KO zebrafish was lower than that of *mTOR*-WT zebrafish at 48 hpa through JC-1 staining (Fig. 5H-I). Therefore, we speculated that *mTOR* may promote mitochondrial fission to regulate fin regeneration.

Following this, we conducted whole ISH assays to assess the expression of genes associated with mitochondrial fission. It was observed that the expression level of mitochondrial dynamin 1-like (*dnm1l*), mitochondrial primase and polymerase (*primpol*), and mitochondrial genome maintenance exonuclease 1 (*mgme1*) was decreased compared to the *mTOR*-WT group (Fig. 5J, Supplementary Fig. 3C-D). Meanwhile, the mRNA expression of *dnm1l*, *primpol* and *mgme1* was consistent with the results of ISH (Fig. 5K, Supplementary Fig. 3E-F). In conclusion, these finding indicated that *mTOR* may promote mitochondrial fission to modulate fin regeneration.

### Ca^2+^ signaling was attenuated in *mTOR*-KO zebrafish larvae after fin amputation

**Fig. 6:**
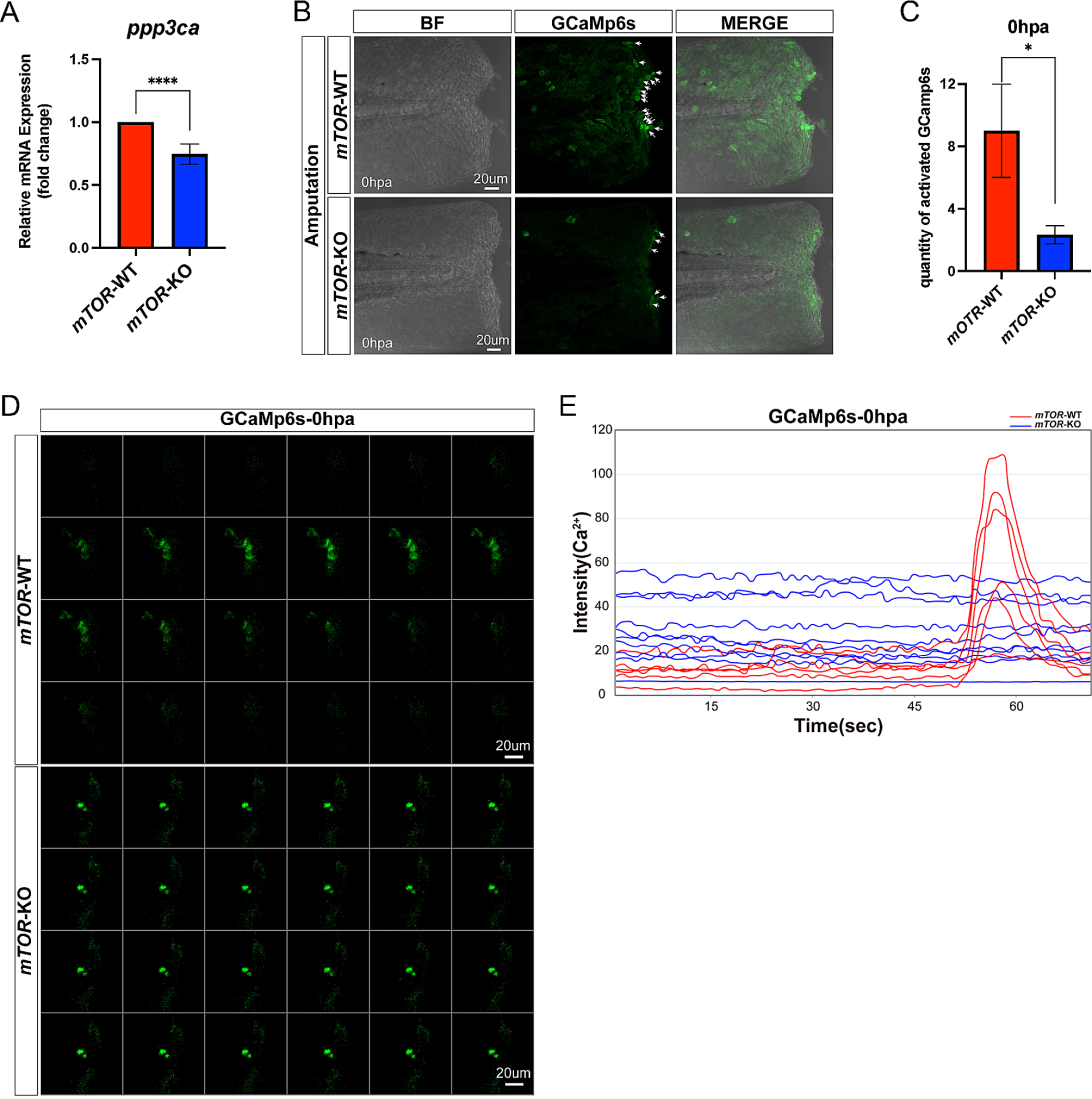
Ca^2+^ signaling was attenuated in *mTOR*-KO zebrafish larvae after fin amputation. (**A**) Comparison of mRNA expression level of *ppp3ca* between *mTOR*-WT and *mTOR*-KO larval zebrafish fin. (**B-C**) Analysis of Ca^2+^ signaling between *mTOR*-WT and *mTOR*-KO larval zebrafish fin after amputation. (**D-E**) Real-time in vivo images showing Ca^2+^ signaling between *mTOR*-WT and *mTOR*-KO larval zebrafish fin after amputation. *P > 0.05, ****P < 0.0001

Studies have shown that *dnm1l* can promote mitochondria fission [17], depending on its dephosphorylation state at Ser637 site [18]. Calcineurin regulates its dephosphorylation at Ser637 site [19], and activation of calcineurin requires CaM [20]. CaM can sense intracellular Ca^2+^. Elevated Ca^2+^ signals bind to and activate CaM [21]. Numerous studies have revealed that intracellular calcium concentration could influence *mTOR* signaling transduction [22–24]. To determine whether *mTOR* regulates mitochondrial functions through Ca^2+^ signaling, we first measured the expression of protein phosphatase 3, catalytic subunit, alpha isozyme (*ppp3ca*, Calcineurin related genes) after fin amputation using the TG(β-actin: GCaMp6s) fish line. Results showed that the mRNA expression level of *ppp3ca* in the *mTOR*-KO group was significantly lower than that in the *mTOR*-WT group (Fig. 6A). Moreover, Ca^2+^ in the tail fin between *mTOR*-WT and *mTOR*-KO group was similar to that at 3 dpf (Supplementary Fig. 4A-B). Ca^2+^ signals in the *mTOR*-WT group were strongly activated within minutes after amputation, whereas Ca^2+^ in the *mTOR*-KO group remained at a low level (Fig. 6B-C). Real-time in vivo imaging observations showed that the Ca^2+^ signaling was continuously suppressed in the *mTOR*-KO group, whereas Ca^2+^ was unaffected in the *mTOR*-WT group (Fig. 4D-E, Supplementary Fig. 4C-D).

These results verified the regulatory relationship between *mTOR* and Ca^2+^ signaling. Upon fin amputation, a disturbance in the equilibrium of the intra- and extracellular environments ensued. This disturbance led to the opening of the Ca^2+^ channel on the cellular membrane, facilitating the influx of Ca^2+^ and subsequently elevating the concentration of Ca^2+^ in the cytoplasm, thereby triggering the activation of CaM. This intricately orchestrated process relied on the involvement of *mTOR*, which in turn spurred the downstream *dnm1l* activation, consequently promoting the mitochondrial fission response essential for fin regeneration.

## Discussion

**Fig. 7:**
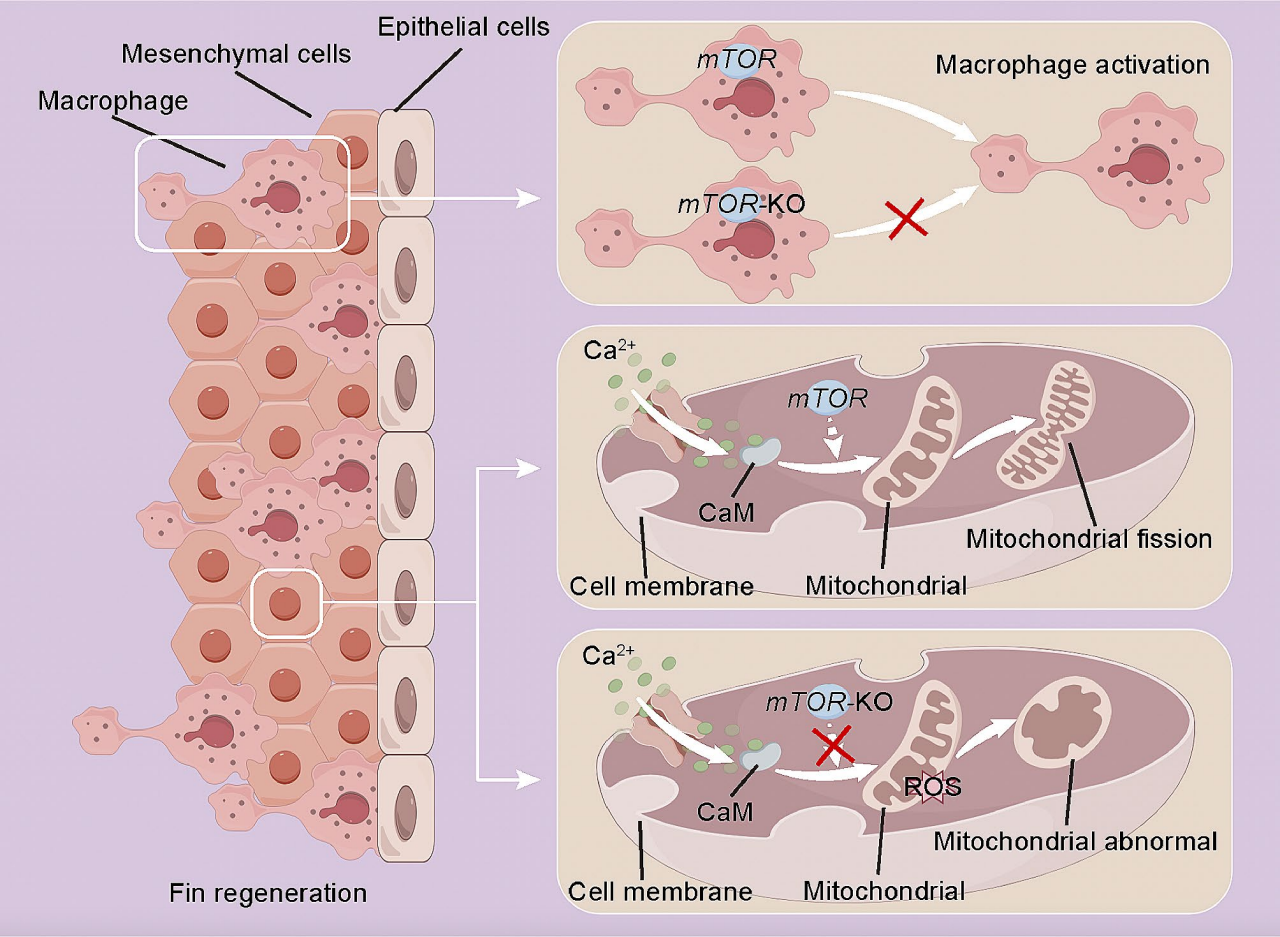
Schematic illustration of the mechanism by which *mTOR* mutation disrupts larval zebrafish tail fin regeneration via regulating proliferation of blastema cells and mitochondrial functions

Strategies for promoting tissues or organs and restore their original functions after injuries have been extensively investigated. In this study, we established a larval zebrafish tail fin amputation model to explore the role of *mTOR* in regeneration. Results showed that the *mTOR* signaling was activated after fin amputation and *mTOR* promoted fin regeneration by regulating mitochondrial fission and increasing epithelial and mesenchymal cells proliferation. (Fig. 7).

*mTOR* participates in a wide range of physiological and pathological processes, indicating its diverse functions. *mTOR* has been shown to play a significant role in the regeneration of many tissues and organs [25]. In neuronal regeneration, *mTOR* promotes the regeneration of retinal ganglion cells. Furthermore, in spinal cord hemisection injury regeneration, *mTOR* facilitates the regeneration of corticospinal tract fibers post-spinal cord injury. For skeletal muscle regeneration, *mTOR* stimulates the activation and proliferation of satellite cells, further differentiating into myoblasts and promoting myoblast fusion to form muscle fibers. In a severe liver injury regeneration model, liver regeneration occurs through the transdifferentiation of biliary epithelial cells, during which *mTOR* regulates the proliferation of biliary epithelial cells and the formation of bipotent progenitor cells [25]. Similarly, unique regulatory mechanisms of *mTOR* may exist in fin regeneration, necessitating the establishment of relevant models for further investigation. The molecular mechanisms of fin regeneration are similar between adult zebrafish and zebrafish larvae [3]. Therefore, this study aimed to investigate the regeneration process and mechanisms of this relatively simple structure in zebrafish larvae. Here, results confirmed that *mTOR* played a role in the regeneration process of zebrafish larvae after fin amputation. Interestingly, *mTOR* did not affect cell apoptosis and differentiation in our study. We postulated that the zebrafish larvae may be at an early stage of development and had not yet reached the stage of differentiation. At this time, the blastema cells mainly consist of epithelial cells, mesenchymal cells, which are not exactly similar to those of adult zebrafish. In addition, the fin regeneration cycle of adult zebrafish is much longer compared with that of zebrafish larvae, and previous studies only measured the cell survival at 72 hpa.

Studies have found that during the regeneration stage, blastema cells released from the broken end of fin proliferate to form a new fin [3]. Mitochondria exhibit different phenotypes under various physiological and pathological conditions [26]. Mitochondria produce young mitochondria through the fission process, while the old, damaged and irreparable mitochondria are eliminated. Numerous studies have linked the mitochondrial fission process to a series of reactions that can cause pathological phenotypes and cell death [27–29]. In contrast, some recent studies have demonstrated that the mitochondrial fission has positive implications for promoting damage repair [30–32]. This is consistent with our results. There was significant mitochondrial fission after fin amputation in *mTOR*-WT zebrafish larvae, but this phenomenon was significantly inhibited in the *mTOR*-KO larval zebrafish tail fin. Previous studies have shown that *mTOR* regulates mitochondrial dynamics and cell survival through *MTFP1* [33], suggesting a regulatory mechanism between *mTOR* and mitochondria. Analysis of the SMART-seq data revealed the genes associated with altered mitochondria-related functions and targeted the key regulated gene, *dnm1l*. As expected, the expression of *dnm1l*, a pivotal gene that regulates mitochondrial fission, was downregulated in *mTOR*-KO larvae. The expression level of calcineurin which controls *dnm1l* was also down-regulated, indicating that *mTOR* may regulate *dnm1l* by affecting the upstream pathways of calcineurin. After fin amputation, the body mounts a quick response and Ca^2+^ signaling is immediately activated in cells surrounding the injury, acting as a second messenger that transmits the injury signal and initiates regeneration process [34]. Some studies have reported that CaM activates *mTOR* pathway in response to increased intracellular Ca^2+^ level^35^. To further explore whether the effect of *mTOR* on mitochondria involves intracellular Ca^2+^ signaling, we used Ca^2+^ labelled transgenic fish lines to measure Ca^2+^ signaling activity. Results showed that Ca^2+^ signaling activity was significantly inhibited after *mTOR* knockout. Interestingly, we detected the fluorescence emitted by the binding of Ca^2+^ to calmodulin. Therefore, we postulated that *mTOR* was required for Ca^2+^ signaling activation to promote mitochondrial fission.

CaM is a widely expressed EF-hand calcium sensor protein. The conformation of CaM changes from a closed to an open state upon binding with Ca^2+^, exposing a hydrophobic surface that facilitates binding with its target proteins [21, 36]. CaM has been reported to interact with amino acids [37, 38], human vacuolar protein sorting 34 (HVps34) [39], and tuberous sclerosis complex 2 (TSC2) [35] to enhance *mTORC1* activity. Calcium/calmodulin has also been reported to directly bind to *mTOR* through the lysosome-resident calcium channel, transient receptor potential mucolipin 1 (TRPML1) [39]. There is limited research on the relationship between calcium levels and *mTORC2*, but a recent study has indicated that *mTORC2* assembly and activity are regulated by calcium signaling [40]. However, it remains unclear how *mTORC2* perceives calcium ions. Further research in this area will be conducted to elucidate its mechanisms of action.

## Conclusion

This study has demonstrated the multifaceted roles of *mTO*R in zebrafish larval fin regeneration. *mTOR* is activated post-amputation and regulates metabolic processes. Furthermore, *mTOR* can modulate mitochondrial dynamics through calcium signaling to respond to tissue injury. These findings enhance our understanding of the regeneration process.

### Electronic supplementary material

Below is the link to the electronic supplementary material.

Supplementary Material 1

Supplementary Material 2

Supplementary Material 3

Supplementary Material 4

Supplementary Material 5

Supplementary Material 6

## Data Availability

The SMART-seq results are deposited in GEO repositories (GEO accession: GSE242949; https://www.ncbi.nlm.nih.gov/geo/query/acc.cgi?acc=GSE242949).

## Abbreviations

*mTOR*: mechanistic target of rapamycin
*mTOR*-KO: *mTOR* knockout
ISH: in situ hybridization
PFA: paraformaldehyde
hpa: hous post amputation
dpf: days post fertilization
DCFH-DA: Dichloro-dihydro-fluorescein diacetate
TEM: transmission electron microscope
DEGs: differentially expressed genes
*dnm1l*: mitochondrial dynamin 1-like
*primpol*: mitochondrial primase and polymerase
*mgme1*: mitochondrial genome maintenance exonuclease 1
*ppp3ca*: protein phosphatase 3, catalytic subunit, alpha isozyme
ECM: extracellular matrix
DAMPs: damages associated molecular patterns
HVps34: human vacuolar protein sorting 34
TSC2: tuberous sclerosis complex 2
TRPML1: ransient receptor potential mucolipin 1
