# Supplementary material for: mTOR mutation disrupts larval zebrafish tail fin regeneration via regulating proliferation of blastema cells and mitochondrial functions
Source: J Orthop Surg Res. 2024 May 29;19:321. doi: 10.1186/s13018-024-04802-z (PMC11134885; doi:10.1186/s13018-024-04802-z)
Supplement: Supplementary file 1 — Supplementary Material 1 [file 13018_2024_4802_MOESM1_ESM.docx]

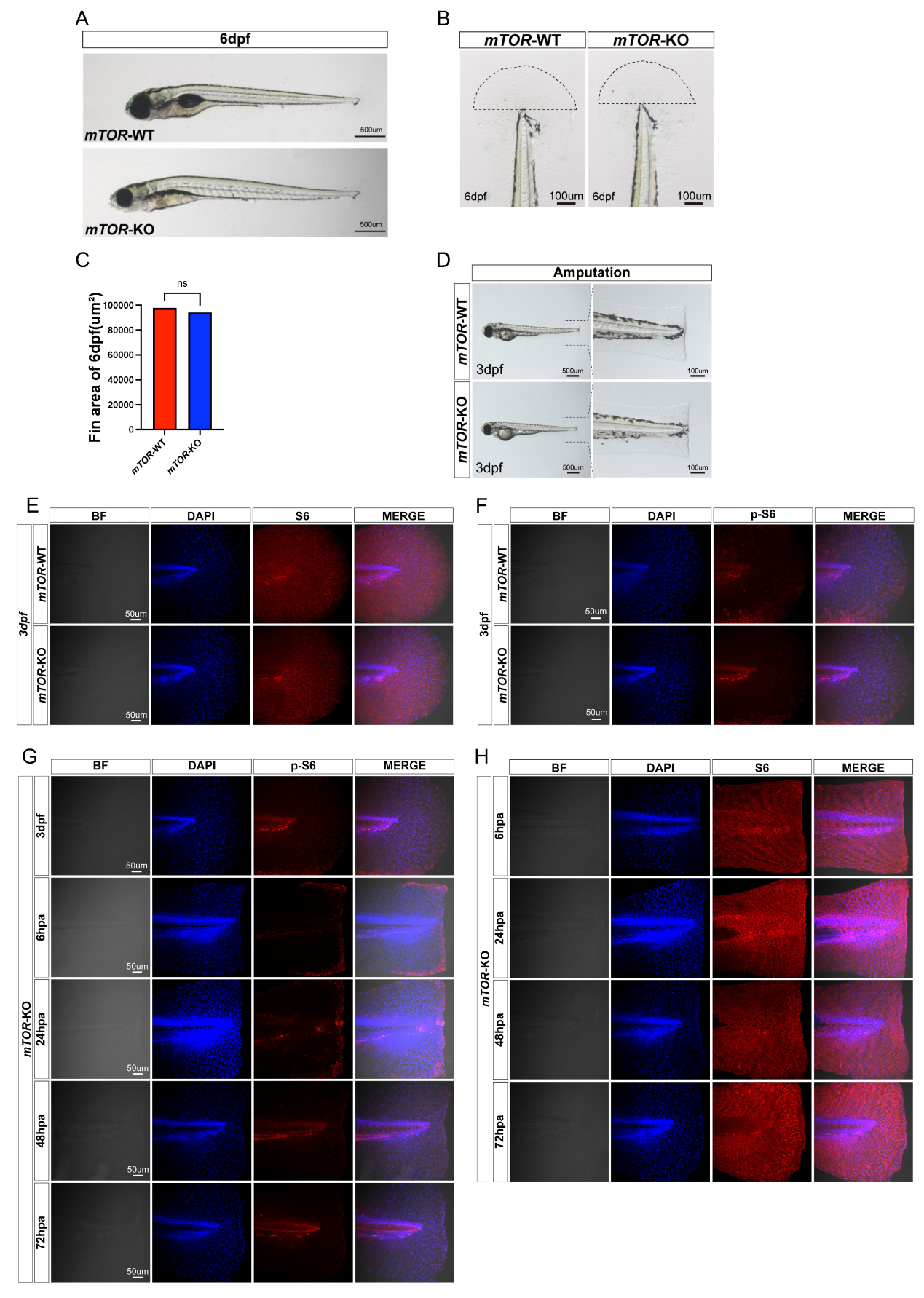


**Supplementary Fig. 1 mTOR knock out didn’t affect caudal fin development. (A)** The morphology of *mTOR*-WT and *mTOR*-KO zebrafish larvae at 6 dpf. **(B-C)** The fin area of un-amputated *mTOR*-WT and *mTOR*-KO zebrafish at 6 dpf. **(D)** Amputation position for fin regeneration model. **(E)** Comparison of S6 expression level between *mTOR*-WT and *mTOR*-KO larval zebrafish tail fin at 3 dpf. **(F)** Comparison of p-S6 expression level between *mTOR*-WT and *mTOR*-KO larval zebrafish tail fin at 3 dpf. **(G)** The expression of p-S6 in *mTOR*-KO zebrafish larvae at different stages following fin amputation. **(H)** The S6 expression levels of *mTOR*-KO zebrafish larvae at different stages after fin amputation. ^ns^ P > 0.05.
