# Supplementary material for: mTOR mutation disrupts larval zebrafish tail fin regeneration via regulating proliferation of blastema cells and mitochondrial functions
Source: J Orthop Surg Res. 2024 May 29;19:321. doi: 10.1186/s13018-024-04802-z (PMC11134885; doi:10.1186/s13018-024-04802-z)
Supplement: Supplementary file 2 — Supplementary Material 2 [file 13018_2024_4802_MOESM2_ESM.docx]

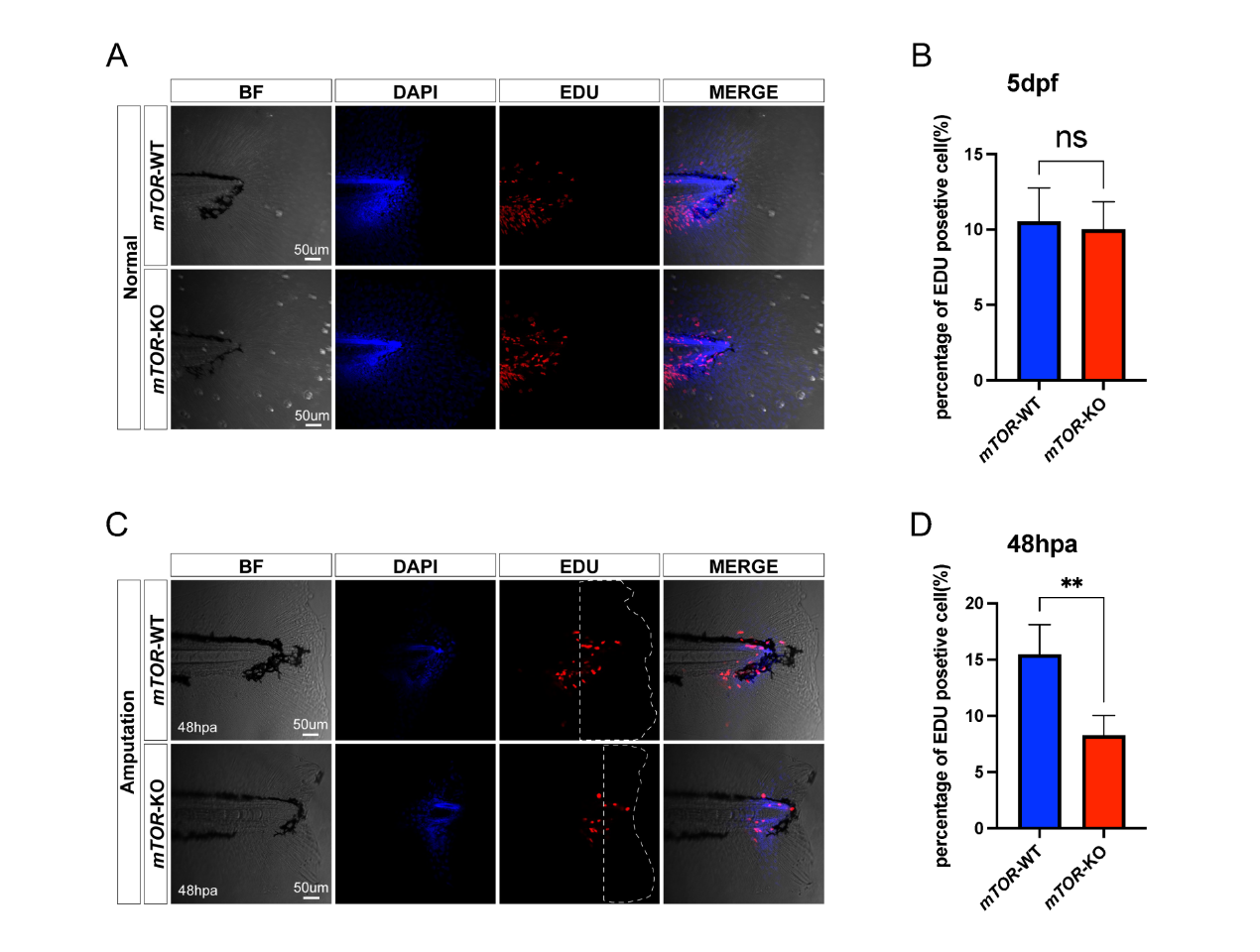


**Supplementary Fig. 2 *mTOR* knock out suppressed cell proliferation after fin amputation. (A-B)** Results of EDU staining for *mTOR*-WT and *mTOR*-KO larval zebrafish tail fin at 5dpf. **(C-D)** EDU staining between *mTOR*-WT and *mTOR*-KO larval zebrafish tail fin at 48 dpa. ^ns^ P > 0.05, **P < 0.01.
