# Supplementary material for: mTOR mutation disrupts larval zebrafish tail fin regeneration via regulating proliferation of blastema cells and mitochondrial functions
Source: J Orthop Surg Res. 2024 May 29;19:321. doi: 10.1186/s13018-024-04802-z (PMC11134885; doi:10.1186/s13018-024-04802-z)
Supplement: Supplementary file 3 — Supplementary Material 3 [file 13018_2024_4802_MOESM3_ESM.docx]

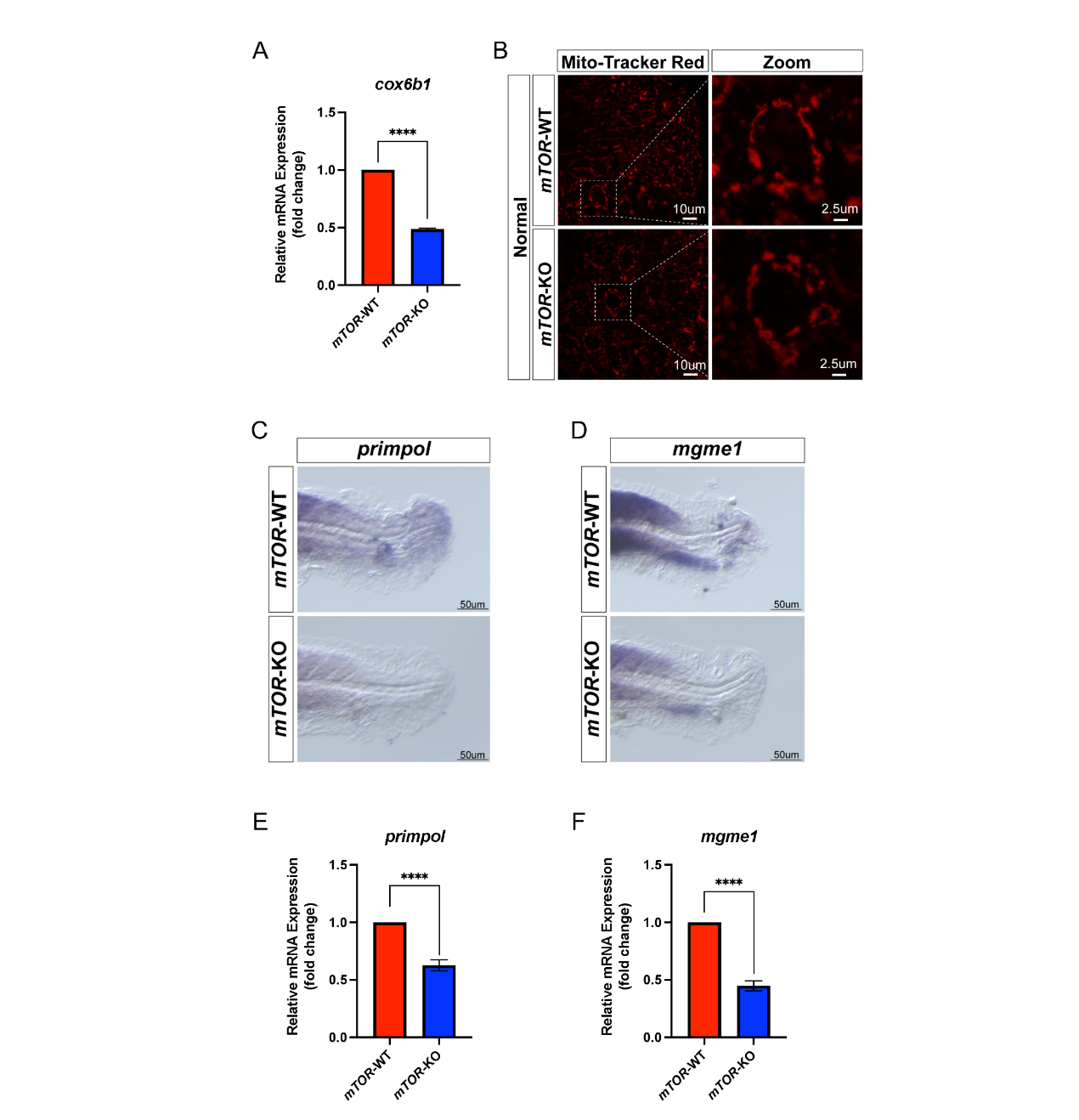


**Supplementary Fig. 3 *mTOR* knockout affected mitochondrial respiratory chain and fission-related functions. (A)** The mRNA expression of *cox6b1* between *mTOR*-WT and *mTOR*-KO larval zebrafish tail fin. **(B)** Mito-Tracker red staining of *mTOR*-WT and *mTOR*-KO larval zebrafish tail fin at 3 dpf. **(C-D)** Location of mitochondrial related genes (*primpol*, *mgme1*) between *mTOR*-WT and *mTOR*-KO larval zebrafish tail fin by in situ hybridization. **(E-F)** mRNA expression levels of *primpol* and *mgme1* between *mTOR*-WT and *mTOR*-KO larval zebrafish tail fin. ****P < 0.0001.
