# Supplementary material for: mTOR mutation disrupts larval zebrafish tail fin regeneration via regulating proliferation of blastema cells and mitochondrial functions
Source: J Orthop Surg Res. 2024 May 29;19:321. doi: 10.1186/s13018-024-04802-z (PMC11134885; doi:10.1186/s13018-024-04802-z)
Supplement: Supplementary file 4 — Supplementary Material 4 [file 13018_2024_4802_MOESM4_ESM.docx]

**
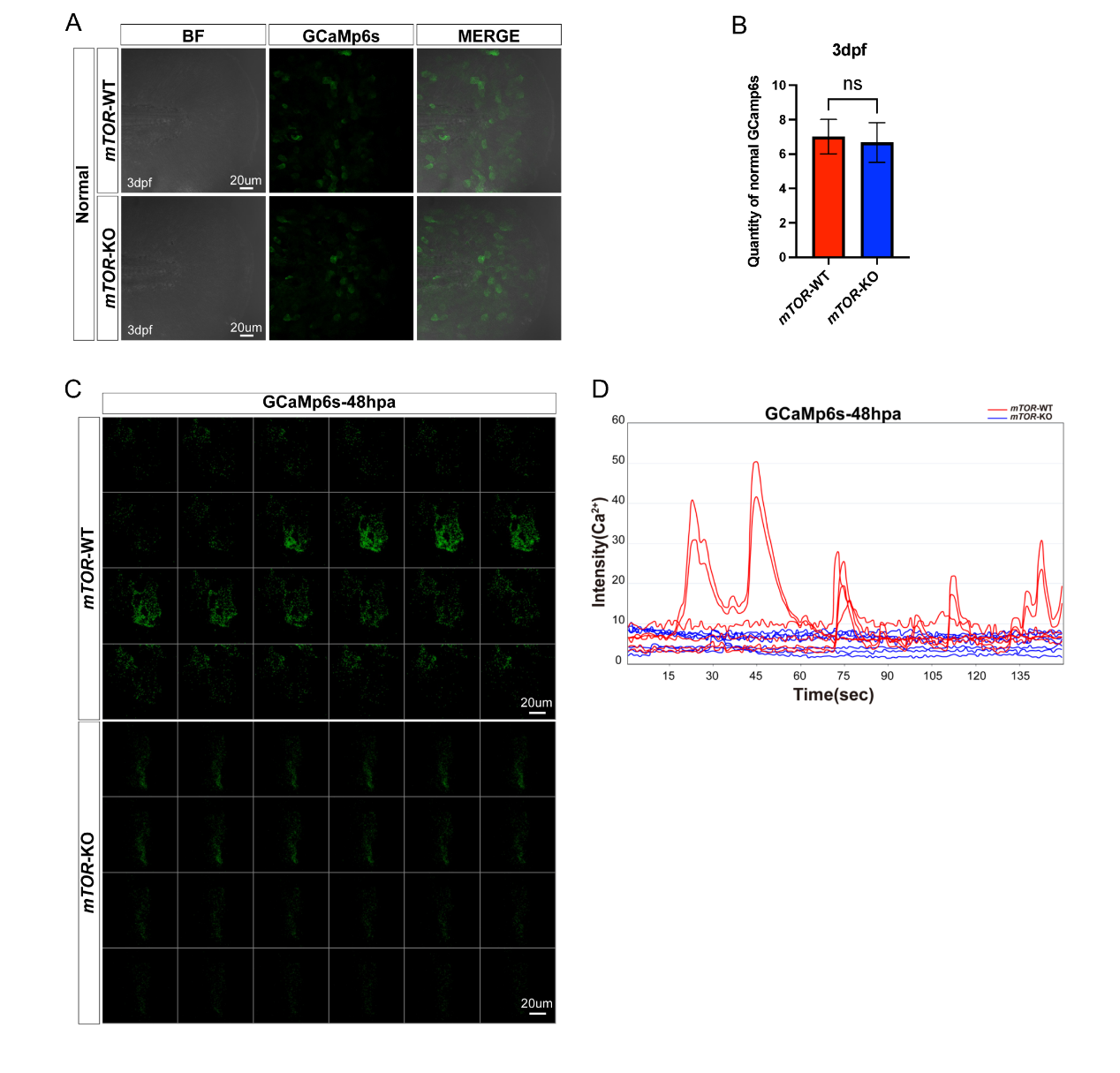
**

**Supplementary Fig. 4 *mTOR* knockout inactivated Ca^2+^ signaling after fin amputation. (A-B)** Comparison of Ca2 + signaling between *mTOR*-WT and *mTOR*-KO larval zebrafish tail fin at 3 dpf. **(C-D)** Real-time in vivo images of Ca^2+^ signaling between *mTOR*-WT and *mTOR*-KO larval zebrafish fin at 48 hpa. ^ns^ P > 0.05.
