# Supplementary material for: mTOR mutation disrupts larval zebrafish tail fin regeneration via regulating proliferation of blastema cells and mitochondrial functions
Source: J Orthop Surg Res. 2024 May 29;19:321. doi: 10.1186/s13018-024-04802-z (PMC11134885; doi:10.1186/s13018-024-04802-z)
Supplement: Supplementary file 5 — Supplementary Material 5 [file 13018_2024_4802_MOESM5_ESM.docx]

**Supplementary Table 1.** Primer sequences used in ISH

| **Gene** | **Species** | **Promoter** | **Sequence (5’ - 3’)** | |
| --- | --- | --- | --- | --- |
| *msx3* | Zebrafish | T7 | Forward | GCAAAAGCGCGGACAAAGAG |
|  |  |  | Reverse | attgtaatacgactcactatagggCGTAGAGTGGTGGAGAACCAAC |
| *junba* | Zebrafish | T7 | Forward | GACTCGTTTCTTTCTGCTTATGG |
|  |  |  | Reverse | attgtaatacgactcactatagggCGACAATAACATCATCACAAACAGG |
| *junbb* | Zebrafish | T7 | Forward | GACTCGTTTCTGTTGGGTTACGG |
|  |  |  | Reverse | attgtaatacgactcactatagggGCCTCCATCTTACTGGTCAGC |
| *mvp* | Zebrafish | T7 | Forward | GGCCCTCTCACCTACATACG |
|  |  |  | Reverse | attgtaatacgactcactatagggCGTTCTCCAGATGCTCACCG |
| *ilf2* | Zebrafish | T7 | Forward | GGCCTTTCCTCGAGTGAAGC |
|  |  |  | Reverse | attgtaatacgactcactatagggGCCCAAGATCTTCCGGTATCC |
| *fn1b* | Zebrafish | T7 | Forward | GGTATATGGTGGTAACTCTGGTGG |
|  |  |  | Reverse | attgtaatacgactcactatagggGCCTTCATACGTCAAACCTGC |
| *cdk1* | Zebrafish | T7 | Forward | CTGGCAGATTTCGGCTTAGCC |
|  |  |  | Reverse | CTTATAGTCTGGCAGAGACTCAACATCTG |
| *dnm1l* | Zebrafish | T7 | Forward | GCGGGAAGAGTTCAGTTTTGG |
|  |  |  | Reverse | attgtaatacgactcactatagggGCTGACAGAACGTTGATACGG |
| *primpol* | Zebrafish | T7 | Forward | CGTATGTGAGAAGCTAGAGGAGG |
|  |  |  | Reverse | attgtaatacgactcactatagggGCTCGTCTGTTAAATAAGCCTGATC |
| *mgme1* | Zebrafish | T7 | Forward | GCAGGTAACGTTATTGAGGCAG |
|  |  |  | Reverse | attgtaatacgactcactatagggGCAGGTGAACCGTCTTTGTAG |
