# Supplementary material for: mTOR mutation disrupts larval zebrafish tail fin regeneration via regulating proliferation of blastema cells and mitochondrial functions
Source: J Orthop Surg Res. 2024 May 29;19:321. doi: 10.1186/s13018-024-04802-z (PMC11134885; doi:10.1186/s13018-024-04802-z)
Supplement: Supplementary file 6 — Supplementary Material 6 [file 13018_2024_4802_MOESM6_ESM.docx]

**Supplementary Table 2.** Primer sequences used in RT-qPCR

| **Gene** | **Species** | **Sequence (5’ - 3’)** | |
| --- | --- | --- | --- |
| *ccna2* | Zebrafish | Forward | GCGTGCTCCAAGAAAGCACCTTTA |
|  |  | Reverse | TTTCCCGCAAATGCGTGTG |
| *ccnb1* | Zebrafish | Forward | TGTGATGCAGCATATTGCCAAA |
|  |  | Reverse | GGCAGTGAAGAAATCCGTAAAATAAA |
| *ccnd1* | Zebrafish | Forward | CTGGACAGGTTTTTATCTGTGGAGCC |
|  |  | Reverse | GCTTGGAGCTCTGATGTATAGGCAGT |
| *cdk1* | Zebrafish | Forward | CTGGCAGATTTCGGCTTAGCC |
|  |  | Reverse | CTTATAGTCTGGCAGAGACTCAACATCTG |
| *cox6b1* | Zebrafish | Forward | CAAAGGTGTGGATACAGCC |
|  |  | Reverse | CATTTCTCGATCCAGGACAG |
| *dnm1l* | Zebrafish | Forward | TCACCAATGAGATGGTTCAC |
|  |  | Reverse | AAGCATCAGCAAAGTCTGG |
| *primpol* | Zebrafish | Forward | ATGGCATTATTACAGCACTCAC |
|  |  | Reverse | TAGAGTTTGCAGACAGCCC |
| *mgme1* | Zebrafish | Forward | GCTACCAGGTAGATAATGGCT |
|  |  | Reverse | GCTTGGAGTTCAGAAAGTGAG |
